# Wheat plant height locus *RHT25* encodes a PLATZ transcription factor that interacts with DELLA (RHT1)

**DOI:** 10.1073/pnas.2300203120

**Published:** 2023-05-01

**Authors:** Junli Zhang, Chengxia Li, Wenjun Zhang, Xiaoqin Zhang, Youngjun Mo, Gabriela E. Tranquilli, Leonardo S. Vanzetti, Jorge Dubcovsky

**Affiliations:** ^a^Department of Plant Sciences, University of California, Davis, CA 95616; ^b^HHMI, Chevy Chase, MD 20815; ^c^Department of Crop Science and Biotechnology, Jeonbuk National University, Jeonju 54896, Republic of Korea; ^d^Instituto Nacional de Tecnología Agropecuaria, Centro de Investigación de Recursos Naturales, Instituto de Recursos Biológicos, Hurlingham 1686 Buenos Aires, Argentina; ^e^Instituto Nacional de Tecnología Agropecuaria, Estación Experimental Agropecuaria, Marcos Juárez, Marcos Juárez 2850 Córdoba, Argentina; ^f^Consejo Nacional de Investigaciones Científicas y Técnicas, Buenos Aires C1425FQB, Argentina

**Keywords:** wheat, plant height, PLATZ, DELLA, natural variation

## Abstract

Plant height is an important agronomic trait with a significant impact on grain yield, as demonstrated by the positive effect of the *REDUCED HEIGHT* (*RHT*) dwarfing alleles (*Rht1b*) on lodging and harvest index in the “Green Revolution” wheat varieties. However, these gibberellic acid (GA)-insensitive alleles also reduce coleoptile length, biomass production, and yield potential in some environments, triggering the search for alternative GA-sensitive dwarfing genes. Here we report the identification, validation, and characterization of the gene underlying the GA-sensitive dwarfing locus *RHT25* in wheat. This gene, designated as *PLATZ-A1* (*TraesCS6A02G156600*), is expressed mainly in the elongating stem and developing spike and encodes a plant-specific AT-rich sequence- and zinc-binding protein (PLATZ). Natural and induced loss-of-function mutations in *PLATZ-A1* reduce plant height and its overexpression increases plant height, demonstrating that *PLATZ-A1* is the causative gene of *RHT25. PLATZ-A1* and *RHT1* show a significant genetic interaction on plant height, and their encoded proteins interact with each other in yeast and wheat protoplasts. These results suggest that PLATZ1 can modulate the effect of DELLA on wheat plant height. We identified four natural truncation mutations and one promoter insertion in *PLATZ-A1* that are more frequent in modern varieties than in landraces, suggesting positive selection during wheat breeding. These mutations can be used to fine-tune wheat plant height and, in combination with other GA-sensitive dwarfing genes, to replace the GA-insensitive *Rht1b* alleles and search for grain yield improvements beyond those of the Green Revolution varieties.

## Significance

We have identified and characterized a previously unknown gene controlling plant height in wheat and named it *PLATZ1*. Mutations in *PLATZ1* reduce plant height while its overexpression results in taller plants. *PLATZ1* is expressed mainly in elongating stems and developing spikes and shows a significant genetic interaction on plant height with the “Green Revolution” dwarfing gene *REDUCED HEIGHT 1* (*RHT1*). We identified five natural mutations in the A genome copy of *PLATZ1* in common wheat that have been favored during breeding, suggesting an overall positive effect on wheat performance. These mutations can be used to fine-tune wheat plant height and, eventually, to replace the *RHT1* dwarfing alleles that impose limitations on planting depth and grain yield potential in some environments.

Wheat is a critical crop for global food security and continuous increases in grain yield are required to feed a growing human population that recently surpassed 8 billion people. The large increases in wheat grain yield obtained by the introduction of the *REDUCED HEIGHT* (*RHT*) dwarfing alleles *Rht-B1b* and *Rht-D1b* during the “Green Revolution” demonstrated that optimizing plant height is critical to reduce lodging and improve harvest index (1). *RHT1* encodes a protein designated DELLA, which is a critical component of the gibberellin (GA) growth-stimulating pathway (2). GA binds the GID1 (GIBBERELLIN INSENSITIVE DWARF1) receptor altering its conformation and favoring its interaction with the N-terminal region of DELLA, which promotes its subsequent degradation, the release of DELLA-repressed targets, and GA-mediated growth responses (3, 4). Although both *Rht-B1b* and *Rht-D1b* alleles carry premature stop codons in the N-terminal region, DELLA proteins translation is reinitiated after the GID1-GA-binding region resulting in GA-insensitive constitutively active repressors (5).

Wheat plants that carry the *Rht-B1b* and *Rht-D1b* alleles accumulate the growth-repressing DELLA proteins, which reduces the stimulating effect of the GA hormone on plant growth (6). As a result, semidwarf varieties with the *Rht1b* allele have shorter coleoptiles and reduced above-ground biomass (7). The shorter coleoptiles limit planting depth and access to soil moisture, whereas the reduced biomass can be detrimental in water-limited environments (8). Reduced biomass can also limit grain yield potential in optimum environments, particularly in varieties with excess “sink” capacity (e.g., grain number and size) where a limited “source” (e.g., biomass) can become the limiting factor. Significant progress has been made in recent years in the identification of new alleles for increased grain number (9–11) and grain size in wheat (12), so further increases in biomass are required to fill this extra “sink” capacity. This has triggered research interest in identifying and testing new GA-sensitive *RHT* genes to replace the GA-insensitive *RHT1* alleles.

The four GA-sensitive wheat *RHT* genes cloned and validated so far show a diversity of gene classes and mechanisms. An *RNase H-like* gene encoding a protein with a zinc finger BED-type motif (*RNHL-D1*) was recently found linked to *RHT8*. Loss-of-function mutations in the two linked copies of this gene (*TraesCSU02G024900* in RefSeq v1.1) were found in multiple varieties carrying the *Rht8* dwarfing allele. Akakomugi, which is the source of initial *Rht8* allele, has a different nonfunctional allele of *RNHL-D1* (13, 14). A closely linked but separate locus associated with wheat plant height was mapped in other varieties derived from Akakomugi (15), suggesting the possibility of two linked dwarfing alleles associated with *Rht8*. The semidwarf allele *Rht-B13b* (MSTRG.55039) encodes an autoactive nucleotide-binding site/leucine-rich repeat (NB-LRR) protein that causes reduced height due to transcriptional upregulation of pathogenesis-related genes (16). The other two cloned *RHT* genes affect the expression of GA2-oxidase genes that regulate plant height by inactivating endogenous bioactive GA isoforms. The *RHT12* allele for reduced plant height is associated with increased expression of *GA2ox-A14* (17, 18), whereas the linked *RHT14*, *RHT18*, and *RHT24* loci are all associated with higher expression of *GA2ox-A9* and reduction of bioactive GA levels in the stems (19, 20).

We previously mapped *RHT25* (*QHt.ucw-6AS*) within a 0.2 cM interval on the short arm of chromosome 6A using a population derived from the cross between common wheat lines UC1110 and PI 610750 (21). This genetic interval defined a 4.3 Mb region (144.0 to 148.3 Mb) including 26 high-confidence annotated genes in the Chinese Spring (CS) RefSeq v1.1, but the causative gene was not identified (21). In this study, we report the identification and validation of the *RHT25* causative gene and its interactions with DELLA. We also describe *RHT25* natural variation and the potential use of these natural alleles to fine-tune plant height in wheat improvement and to develop GA-sensitive semidwarf wheat varieties.

## Results

### Identification of the *RHT25* Candidate Gene.

To identify the most likely candidate gene for the *RHT25* locus, we took advantage of several mapping populations whose parental lines were previously genotyped by exome capture (22) and characterized for plant height. These included the UC1110 x PI 610750 population used for the initial mapping of *RHT25* (21) and eight recombinant inbred line (RIL) populations generated by crossing the variety “Berkut” to different parents (Table 1). These eight populations are part of a nested association mapping (NAM) population previously evaluated for plant height in multiple environments (23, 24).

**Table 1.** Polymorphisms among parents of the NAM populations and the original population used to map *RHT25* (UC1110 x PI 610750). Only polymorphisms within the coding regions and splice sites of genes located in the *RHT25* candidate gene region are presented here

Gene ID	SNP 6AS	Effect	UC1110	PI 610750 Berkut	RAC875	UC1036 Dharwar Dry PI 70613	CItr 7635^*^ PBW343 RSI5^*^	P515HP^†^
RefSeq v1.1	Coordinate	*Rht25* allele:	Short	Tall	Tall	Tall	Tall	Short
*TraesCS6A*		Haplotype:	H1	H2	H3	H4	H5	H6
*02G155900*	144717100	L215L	G	G	A	G	G	G
***02G156600***	145651904/16	Frame shift	13del^‡^	WT	WT	WT	WT	WT
***02G156600***	145651971	Splice site	T	T	T	T	T	C
*02G156700*	145739155	R12C	A	G	G	G	G	G
*02G157200*	146501774	A44A	C	C	C	C	C	A
02G157500	147305219	W165R	A	A	A	A	A	G

^*^RSI5 and CItr 7635 have a promoter insertion.

^†^The SNPs specific for P515HP are underlined.

^‡^The 13-bp deletion was tested in all accessions with molecular markers described in Dataset S2. For the *Rht25* alleles, Tall = no significant differences in plant height with PI 610750 or Berkut, and short = significant differences with the previous two parents. A complete list of the SNPs and the haplotypes in this region is available in Dataset S1. The prioritized candidate gene *TraesCS6A02G156600* is indicated in bold.

In the original population used to map *RHT25*, UC1110 was associated with the dwarfing allele and PI 610750 with the allele for increased plant height, which is partially dominant over the dwarfing allele (21). Among the eight NAM populations, we only detected significant effects for plant height associated with the *RHT25* locus in the Berkut x Patwin-515HP (henceforth P515HP) population, where P515HP carries the allele for reduced plant height and Berkut the allele for increased plant height. The absence of significant differences in plant height associated with the *RHT25* region in the other seven NAM populations suggested that their parental lines carry the same allele as Berkut (Table 1). Using exome capture data for the parental lines (https://triticeaetoolbox.org/wheat/), we identified 17 SNPs and 2 indels in the *RHT25* candidate gene region that defined six haplotypes (Dataset S1). Within this region, *TraesCS6A02G156600* was the only gene that showed loss-of-function polymorphisms in the two haplotypes (H1 and H6) associated with the *RHT25* dwarfing allele (Table 1).

In UC1110 (haplotype H1), *TraesCS6A02G156600* has a 13-bp deletion in the third exon (CS 6A: 145,651,904 -145,651,916) that generates a shift in the reading frame and a premature stop codon that alter or eliminate 59% of the predicted amino acids. We developed a marker for this deletion (PlzAF2/PlzAR1, Dataset S2) and confirmed its presence in UC1110 and its absence in all other accessions in Table 1. P515HP (haplotype H6) has a mutation in the acceptor splice site of *TraesCS6A02G156600* second intron (CS 6A 145,651,971), which results in the retention of the second intron and a premature stop codon that eliminates 66% of the predicted amino acids. The other nine accessions showed no polymorphisms in the coding region of *TraesCS6A02G156600*. Moreover, no other gene in the *RHT25* candidate region showed polymorphisms differentiating the two accessions associated with the *RHT25* dwarfing allele from the other accessions carrying the *RHT25* allele associated with tall plants (Table 1). Based on these results, we concluded that *TraesCS6A02G156600* was the most likely candidate gene for *RHT25*, and prioritized it for functional characterization and validation.

### *PLATZ* Phylogeny and Nomenclature.

*TraesCS6A02G156600* encodes a protein previously designated as “plant-specific AT-rich sequence- and zinc-binding protein” (*PLATZ*) (25). A previous phylogenetic study of 62 *PLATZ* genes identified in wheat (*TaPLATZ1* to *TaPLATZ62*) revealed six groups (I to VI) (26), with *TraesCS6A02G156600* (*TaPLATZ34*) included in group III (26). The two previously characterized wheat *PLATZ* genes belong to group I (*TaPLATZ5*, *TraesCS2D02G447400*) (27) and group II (*TaFl3*, *TraesCS3A02G497900*) (28), so no functional information is currently available for wheat genes from group III.

We performed a phylogenetic analysis for the 12 wheat PLATZ proteins from group III (*SI Appendix*, Figs. S1 and S2), identified homeologs, and assigned them identification numbers following the guidelines for wheat genes (Dataset S3). This analysis showed four well-defined clades designated hereafter as PLATZ1 to PLATZ4, with *TraesCS6A02G156600* included in the PLATZ1 homeologous group (*SI Appendix*, Figs. S1 and S2 and Dataset S3). PLATZ1 and PLATZ2 are closely related to each other and to the well-studied PsPLATZ1 (25) and AtORESARA15 (29) proteins from *Pisum sativum* and *Arabidopsis*, respectively (*SI Appendix*, Fig. S1), whereas PLATZ3 and PLATZ4 are more divergent.

### Validation of *PLATZ-A1* Function Using Ethyl Methanesulfonate (EMS) Mutants.

To confirm the effect of *PLATZ1* on wheat plant height, we characterized loss-of-function mutations in both *PLATZ-A1* and *PLATZ-B1* (*TraesCS6B02G282400LC*) homeologs found in the sequenced Kronos EMS population (22). For *PLATZ-A1*, we identified mutant K2557 that carries a mutation in the acceptor splice site of the third intron (RefSeqv1.1 chromosome 6A: 145,651,705). For *PLATZ-B1*, we identified mutant K86 that carries a mutation predicted to change an arginine at position 69 to cysteine (R69C) in the second exon (RefSeqv1.1 chromosome 6B: 207,805,286, *SI Appendix*, Fig. S3*A*). The R69C mutation is located within the PLATZ conserved domain (pfam 04640) and is expected to have a disruptive effect in protein structure and function based on a negative BLOSUM62 (-3) and very low SIFT score (0) (*SI Appendix, Method S3*).

To test the effect of the splice site mutation in *PLATZ-A1*, we sequenced its transcripts from mutant K2557 using primers Platz6A-F2 and Platz6A-R1 (Dataset S2) and found two different transcripts. The first and more abundant variant (*SI Appendix*, Fig. S3*B*) showed retention of the third intron, which generates a premature stop codon and the loss of 46.5% of the predicted amino acids. The less abundant variant uses the alternative second next AG splicing site within exon 4, which results in the elimination of 21 bp and the deletion of amino acids VDHVVEQ between positions 131 and 137. The deleted amino acids are located in a very conserved region of the PLATZ1 and PLATZ2 proteins, and likely affect protein function (*SI Appendix*, Fig. S2).

**Fig. 1.**
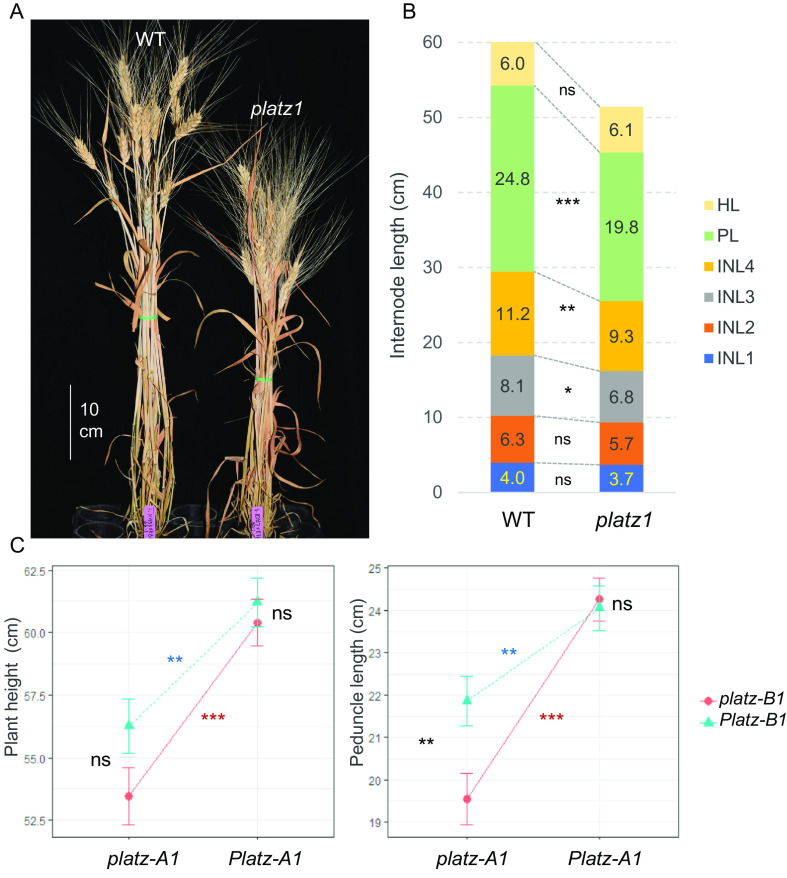
Effect of *PLATZ1* induced mutations on plant height and peduncle length. (*A*) Plant height difference in representative plants. WT = wild type and *platz1* = double-mutant *platz-A1 platz-B1*. (*B*) Differences between wild type and *platz1* in head length (HL), peduncle length (PL) and internodes length (INL). (*C*) Interaction graphs for *PLATZ-A1* and *PLATZ-B1* (nonparallel lines reflect interactions). Error bars are s.e.m. based on n = 7 to 17. ns = not significant, ** = *P* < 0.01, and *** = *P* < 0.001 for simple effect contrasts in the factorial ANOVA (Dataset S4).

We crossed the K2557 and K86 mutants to nonmutagenized Kronos plants five times to reduce background mutations and intercrossed them to generate a BC_4_F_2_ population segregating for mutations in both homeologs. Compared with the wild type, the double mutant (henceforth *platz1*, PI 702421) showed a significant (*P* < 0.0001) reduction in plant height (12.7%), peduncle length (18.7%), and other internodes (Fig. 1 *A* and *B* and Dataset S4). A factorial ANOVA using the two homeologs as factors showed significant effects in plant height and peduncle length (*P* < 0.0001) for *PLATZ-A1*, but marginally nonsignificant effects for *PLATZ-B1* (Dataset S4).

Since the interaction between *PLATZ-A1* and *PLATZ-B1* on peduncle length was significant (*P =* 0.0279, Dataset S4), we analyzed the effects of each homeolog in the wild type and mutant backgrounds of the other homeolog using statistical contrasts (Fig. 1*C*). *PLATZ-A1* showed significant effects on peduncle length under both *PLATZ-B1* backgrounds, but the effects were stronger in the presence of *platz-B1*. Similarly, the *platz-B1* allele showed stronger effects on peduncle length in the presence of *platz-A1* mutant allele (Fig. 1*C*). These results indicate that *PLATZ-A1* and *PLATZ-B1* have redundant roles in the regulation of plant height and peduncle length, with *platz-A1* having a stronger effect on these two traits than *platz-B1*.

### Effect of *PLATZ1* EMS Mutations on Grain Number, Size, and Weight.

The previously published evaluation of the UC1110 x PI 610750 population in the field showed no significant differences in grain number per spike but revealed a small but significant reduction in grain weight associated with the *RHT25* dwarfing allele (21). Therefore, we characterized these two traits in a greenhouse experiment using BC_4_F_2_ sister lines segregating for the four possible homozygous combinations of the *platz-A1* and *platz-B1* mutants. We found no significant differences in the number of grains per spike, but detected a significant decrease in kernel weight and spike grain yield associated with the *platz-A1* mutant, which was stronger in the combined *platz1* mutant (Dataset S4A).

We then evaluated the effect of the induced *platz-A1* and *platz-B1* mutations in the field (*SI Appendix, Method S10*) using BC_4_F_3_ near isogenic lines in semidwarf (*Rht-B1b*) durum wheat varieties Kronos, Desert Gold (PVP 2019-00010), and UC1771-Low Cadmium (UC1771LC). The combined ANOVA including the three lines showed a significant reduction in plant height in the mutants relative to the wild type, with the largest effect in the combined *platz1* mutant (−13.0%), followed by *platz-A1* (−10.5%) and *platz-B1* (−2.5%) (Dataset S4B). The stronger plant height reduction observed in the combined *platz1* mutant relative to the single mutants was reflected in stronger reductions in grain length (−1.4%, *P* < 0.001), grain width (−2.9%, *P* < 0.05), grain area (−2.8%, *P* < 0.001), TKW (−4.1%, *P* < 0.01), and grain yield (−5.5%, *P* < 0.05, Dataset S4B). No significant differences were detected in kernel number per m^2^ for the individual or combined *platz1* mutants (Dataset S4B), suggesting that the reduction in grain size may be the main contributor to the marginally significant reduction in grain yield observed in this experiment. ANOVAs performed separately by variety showed no significant differences in grain yield, and the differences in grain size and weight were significant only in UC1771LC (Dataset S4C). These results suggest that the effects of *PLATZ1* on grain weight can be modulated by genetic background.

### Null Mutants Using CRISPR-Cas9.

To test if the smaller effect of the *platz-B1* mutant was the result of a reduced effect of the single amino acid change, we generated truncation mutants in both *PLATZ1* homeologs using CRISPR-Cas9. We identified one transgenic plant carrying frameshift indels in both *PLATZ-A1* and *PLATZ-B1.* The 1 bp deletion found in *PLATZ-A1* causes a reading frame shift and the truncation of 64% of the protein (*CRplatz-A1*), whereas the 1 bp insertion in *PLATZ-B1* causes a reading frame shift and the loss of 63% of the protein (*CRplatz-B1*, *SI Appendix*, Fig. S3*A*). We crossed this transgenic plant with wild-type Kronos and in the progeny selected plants without the CRISPR-Cas9 vector and carrying both CRISPR-induced mutations.

The F_3_ plants homozygous for the wild type, *CRplatz-A1*, *CRplatz-B1* and combined *CRplatz1* mutant showed similar differences in plant height and peduncle length to those described for the EMS mutants in Fig. 1*C* (*SI Appendix*, Fig. S4 and Dataset S5). In the presence of *platz-A1* mutant allele, the *CRplatz-B1* mutation showed a slightly larger effect on plant height (5.1 cm) and peduncle length (3.4 cm) than the EMS-induced R69C *platz-B1* mutation (plant height = 2.8 cm and peduncle length = 2.3 cm), suggesting that the R69C *platz-B1* mutant may still have some residual activity.

In summary, the combined loss-of-function of the two *PLATZ1* homeologs resulted in significant reductions in plant height and peduncle length (Fig. S4), indicating that this gene is required to maintain the wild-type plant height.

### Transgenic Plants Overexpressing *PLATZ-A1* with and without an HA Tag.

To test if *PLATZ1* was sufficient to induce changes in plant height, we first generated five independent transgenic lines in Kronos overexpressing a fusion of the coding sequence of *PLATZ-A1* and a C-terminal 3xHA tag under the maize *UBIQUITIN* promoter (henceforth, *UBI::PLATZ1-HA*). In leaves of 16-d-old T_1_ transgenic plants, the combined transcript levels of *PLATZ1* transgene and endogenous genes (3 to 16-fold *ACTIN*) were significantly higher than in the nontransgenic sister lines (0.1-fold *ACTIN*, *SI Appendix*, Fig. S5). We also observed significant differences in *PLATZ1* expression in peduncle samples collected close to heading time from selected T_2_ sister lines with and without the transgene from four independent events (*P =* 0.002). In this tissue, transcript levels of *PLATZ1* varied from 43 to 67-fold of *ACTIN* in the presence of the transgene, but were low in the nontransgenic sister lines (0.05 to 0.09-fold *ACTIN*, Dataset S6). In spite of the high transcript levels of *PLATZ1* in the wild-type Kronos plants transformed with the *UBI::PLATZ1-HA* construct, we failed to detect significant differences in plant height in the T_2_ progenies (Dataset S7A).

**Fig. 2.**
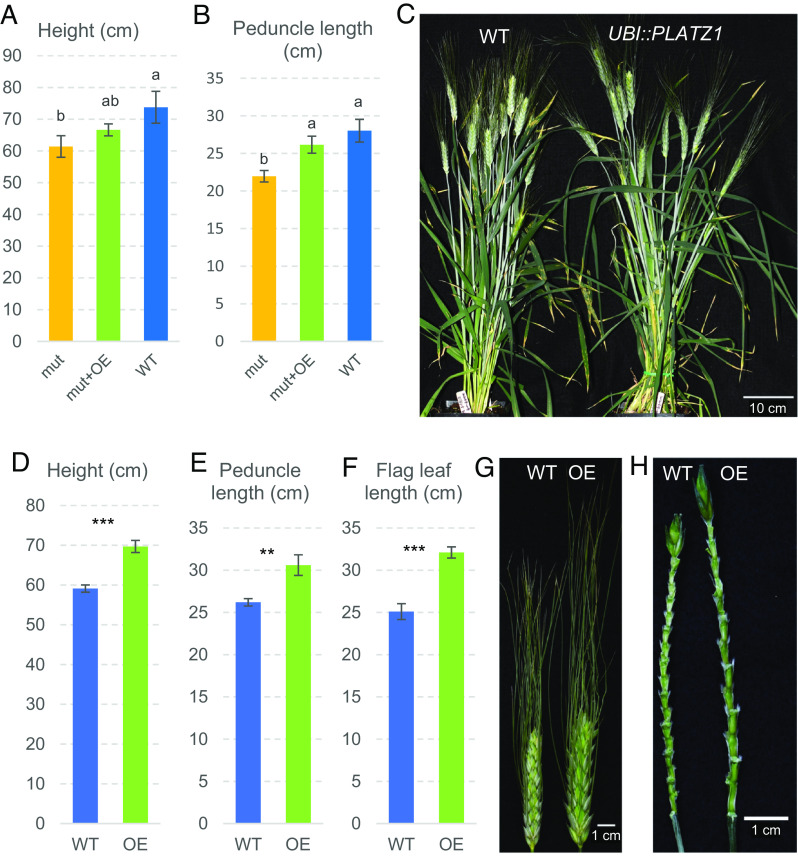
Effect of *UBI::PLATZ1-HA* and *UBI::PLATZ1* transgenes on plant height and peduncle length. (*A* and *B*) Complementation experiment in sister lines carrying the wild-type allele (WT), the *platz1* double mutant (mut), and *platz1* combined with the *UBI::PLATZ1-HA* transgene (mut + OE). Error bars are SEM and different letters above the bars indicate significant differences (*P* = 0.05, n = 6, Dataset S7B). (*C*–*H*) Kronos BC_1_F_1_ plants transformed with the construct *UBI::PLATZ1* without the HA tag (OE n = 8 and WT n = 5, Dataset S7C). (*C*) Selected plants showing differences in plant height. (*D*) Plant height. (*E*) Peduncle length. (*F*) Flag leaf length. ** = *P* < 0.01 and *** = *P* < 0.001. (*G*) Spikes showing fewer but larger spikelets in the *UBI::PLATZ1* transgenic plants (OE) than in the wild type (WT). (*H*) Spikes without the spikelets showing fewer but longer internodes in the plants carrying the *UBI::PLATZ1* transgene (OE) than in the WT. These transgenic plants were mostly sterile.

To test if the lack of differences in plant height was caused by the presence of endogenous *PLATZ1* transcripts or by reduced protein activity caused by the HA tag, we performed two additional experiments. First, we transferred the *UBI::PLATZ1-HA* construct to the Kronos *platz1* combined mutant by crossing. Plants homozygous for the combined *platz1* mutations without the transgene were significantly shorter than the nontransgenic wild type, whereas those carrying the *UBI::PLATZ1-HA* construct showed an intermediate height and peduncle length and no significant differences with the wild type (Fig. 2 *A* and *B* and Dataset S7B). These results indicate that the *UBI::PLATZ1-HA* transgene was able to partially complement the effect of the *platz1* mutant on both peduncle length and plant height.

To test if the presence of the tag was interfering with the function of the PLATZ1 protein, we then transformed Kronos wild-type plants with a *UBI::PLATZ1* construct without the HA-tag. The T_0_ plants were mostly sterile, so we crossed them with wild-type Kronos and analyzed the phenotype in F_1_ and BC_1_F_1_ plants with and without the transgene (Fig. 2 *C*–*H*, and Dataset S7C). Plants carrying the transgene were taller (Fig. 2 *C* and *D*), had longer peduncles (Fig. 2*E*) and longer flag leaves (Fig. 2*F*). Some of the lower leaves were also longer and showed reduced width (Dataset S7C). In addition, the transgenic plants headed later, had spikes with fewer spikelets and longer rachilla internodes (Fig. 2 *G* and *H*). The spikelets were larger (Fig. 2*G*) with significantly longer glumes (Dataset S7C). In summary, the *UBI::PLATZ1* transgenic plants showed more drastic phenotypes in the wild-type Kronos than the same wild-type Kronos plants transformed with the *UBI::PLATZ1:HA*. These results suggest that the HA tag partially interfered with PLATZ1 function, and likely contributed to the lack of differences in plant height in the first experiment.

In summary, the increased plant height of the *UBI::PLATZ1* transgenic plant, the reduced plant height of the *platz1* mutant, and the partial complementation of this reduction by the *UBI::PLATZ1-HA* transgene, demonstrate that *PLATZ-A1* is the causative gene of *RHT25*.

### *PLATZ1* Expression.

Using previously published RNA-seq data, we found that *PLATZ1* transcript levels in hexaploid wheat were the highest in the stem during the early stages of elongation (Z30-Z32, Zadoks’ scale) followed by the early developing spikes (Z32). Transcripts of *PLATZ1* were also observed in the leaves, roots and grains, but at lower levels (30). Transcript levels of *PLATZ-A1* determined by qRT-PCR in different sections of the Kronos elongating peduncle (ear emergence, Zadok 55) were significantly higher in sections including the node and 1 cm immediately above the node than in sections starting 2 cm above the node (Dataset S8 and *SI Appendix*, Fig. S6*C*). In most tissues, *PLATZ1* transcript levels in hexaploid wheat were the highest in the D-genome homeolog, intermediate in the A-genome homeolog, and the lowest in the B-genome homeolog (*SI Appendix*, Fig. S6*A*). We also observed a higher abundance of *PLATZ-A1* compared to *PLATZ-B1* transcripts at different stages of spike development in RNA-seq data for tetraploid wheat Kronos (Fig. S6*B*) (31).

**Fig. 3.**
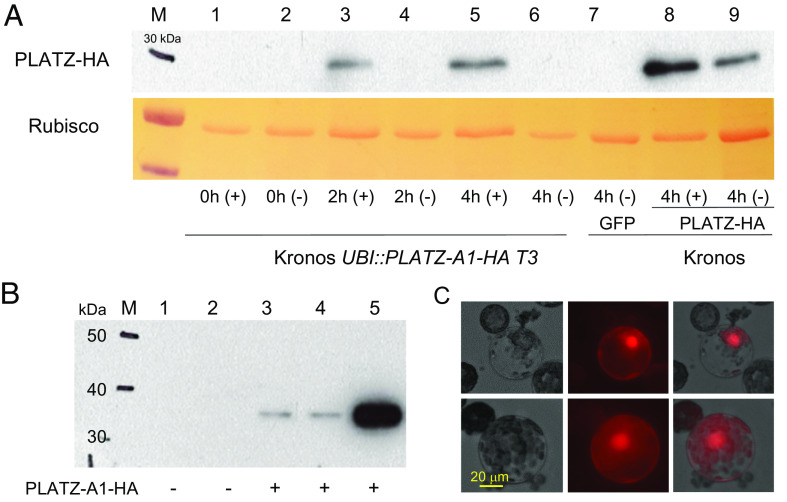
Western blots of PLATZ1-HA using an anti-HA antibody. (*A*) Lanes 1 to 6 are protoplasts prepared from T_3_ transgenic *UBI::PLATZ1-HA* plants treated with either 50 μM MG132 solution in DMSO (+) or only DMSO (−). Protein was extracted 0 h, 2 h, and 4 h after treatment. Lanes 7 to 9 are Kronos protoplasts transiently transformed with *UBI::GFP* (7) or *UBI::PLATZ1:HA* (8–9) with (8) or without MG132 (9). Protein was extracted 4 h after treatment. (*B*) Western blots of proteins extracted from the shoot apical meristem (SAM) and stems. Lanes 1 and 2 are from nontransgenic Kronos plants (negative control), lanes 3 and 4 are from a Kronos *UBI::PLATZ1-HA* T_3_ plant and lane 5 is from transiently transformed Kronos protoplasts (positive control). Lanes 1 and 3 include the SAM and the nodes below collected before elongation (fourth leaf stage, Waddington scale 3.0). Lanes 2 and 4 are from stems collected at the early elongation stage. The expected size of the PLATZ1-HA protein is 32 kDa. (*C*) Nuclear localization of an mCherry-PLATZ-A1 chimera.

We then explored the accumulation profile of the protein encoded by the *UBI::PLATZ1-HA* transgene. First, we performed transient expression in Kronos protoplasts and detected abundant levels of PLATZ1-HA protein in western blots using an anti-HA antibody (Fig. 3*A*, lane 9). However, when we extracted total proteins from the second fully expanded leaf of Kronos T_3_ transgenic plants at the 3-leaf stage, we were unable to detect the protein encoded by the *UBI::PLATZ1-HA* transgene (Fig. 3*A*, lanes 1 and 2), despite high transcript levels (*SI Appendix*, Fig. S5 and Dataset S6).

To test if protein degradation was responsible for our inability to detect the PLATZ1-HA protein in leaves, we treated protoplasts extracted from leaves of the transgenic *UBI::PLATZ1-HA* plants with the proteasome-inhibitor MG132 in solution in DMSO. DMSO without MG132 was used as a negative control. Two hours after the treatment, we detected the PLATZ1-HA protein in the protoplasts treated with MG132 but not in those treated only with DMSO, and the signal became stronger 4 h after the treatment (Fig. 3*A*). These results suggest that the PLATZ1-HA protein is rapidly degraded in the leaves through the proteasome pathway. In control leaf protoplasts from the nontransgenic Kronos plants transiently transformed with *UBI::PLATZ1-HA*, protein levels were higher in the presence of MG132 than in its absence (Fig. 3*A*, lanes 8 and 9), suggesting that the protein was also degraded by the proteasome in the protoplasts. Our western blot analyses detected much higher levels of PLATZ1-HA protein in the transiently transformed protoplasts than in the leaves (both in the presence and absence of MG132), which is likely due to the high concentration of plasmid DNA used for protoplast transfection.

Since the endogenous *PLATZ1* gene is expressed at higher levels in the elongating stems than in the leaves (*SI Appendix*, Fig. S6*A*), we tested if we could detect the PLATZ1-HA protein in the shoot apical meristem (SAM) and stems of the *UBI::PLATZ1-HA* transgenic plants in the absence of MG132. In samples extracted from the SAM and the stem nodes before elongation and in a later stage from early elongating nodes, we detected PLATZ1-HA in the *UBI::PLATZ1-HA* transgenic plants but not in the nontransgenic negative controls (Fig. 3*B*).

Finally, we expressed a *p2xCa35S:*:*mCherry-PLATZ* chimera in the Kronos wheat protoplast to determine its subcellular localization. The PLATZ1 proteins were mainly located in the nucleus (Fig. 3*C*), an expected subcellular localization for a transcription factor.

### Genetic Interactions between *PLATZ1* and *DELLA* on Plant Height and Physical Interactions between Their Encoded Proteins.

To test the genetic interaction between *PLATZ-A1* (*RHT25*) and *RHT1* on wheat plant height, we crossed the *platz-A1* mutant with a tall Kronos near-isogenic line in which the *Rht-B1b* dwarfing allele was replaced by the *Rht-B1a* allele. We selected plants homozygous for the four possible allele combinations in the F_2_ progeny and evaluated the F_3_ progeny for plant height and peduncle length (Dataset S9). Factorial ANOVAs for plant height and peduncle length showed significant effects for both *PLATZ-A1* and *RHT1*, significant interactions between the two genes for plant height (*P =* 0.011), and marginally nonsignificant interaction for peduncle length (*P* = 0.061, Dataset S9).

**Fig. 4.**
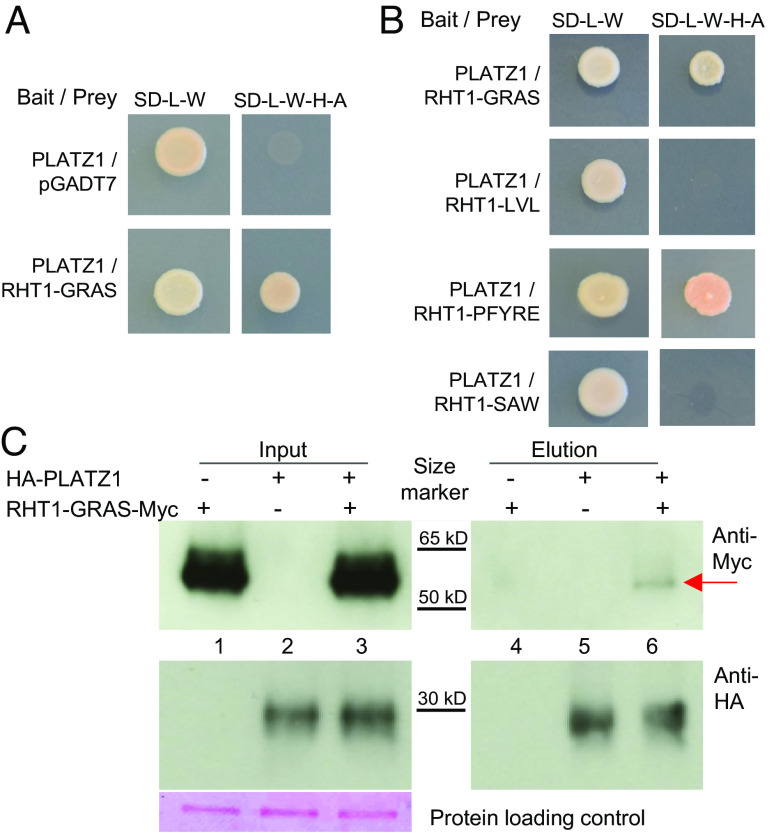
Physical interactions between PLATZ-A1 and DELLA (RHT1). (*A*) Yeast-two-hybrid (Y2H) assays between DELLA and PLATZ-A1. (*B*) Y2H between the complete PLATZ-A1 protein and three subdomains of the DELLA-GRAS protein (LVL, PFYRE, and SAW). (*C*) Co-IP assay between PLATZ-A1 and RHT1-GRAS in wheat protoplasts. The pull-down assay was carried out with anti-HA magnetic beads. Ponceau S staining was used as loading control. The red arrow indicates the RHT1-GRAS-Myc protein coprecipitated with HA-PLATZ1. The black lines indicate the position of the protein size ladder.

We then tested if the significant genetic interaction between *RHT25* and *RHT1* (*DELLA*) on plant height was associated with a physical interaction between their encoded proteins using yeast-two-hybrid (Y2H) assays. We used a truncated DELLA protein carrying only the C-terminal GRAS domain (RHT1-GRAS) because the full-length protein showed autoactivation in Y2H assays. PLATZ-A1 showed a positive interaction with RHT1-GRAS (Fig. 4*A*). We further dissected the RHT1-GRAS domain into three subdomains and evaluated their physical interactions with PLATZ1. We observed a positive interaction between PLATZ1 and the PFYRE subdomain, but no interaction with the LVL and SAW subdomains in the Y2H assays (Fig. 4*B*).

A coimmunoprecipitation (Co-IP) experiment using Kronos leaf protoplasts validated the physical interaction between PLATZ-A1 and DELLA-GRAS in vivo. We cotransformed HA-PLATZ1 and RHT1-GRAS-Myc into Kronos protoplasts and, after immunoprecipitation with anti-HA beads, we detected a weak signal for RHT1-GRAS-Myc with the anti-Myc antibody (Fig. 4*C*).

**Fig. 5.**
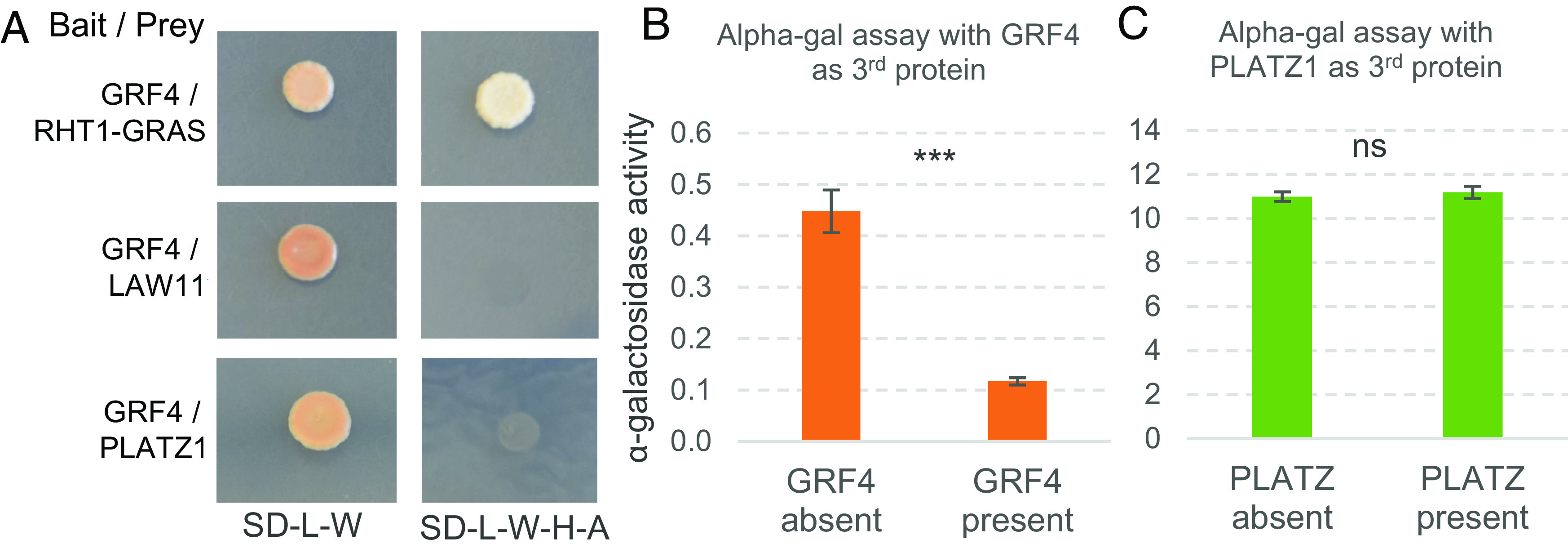
Yeast-three-hybrid (Y3H) assays between GRF4, DELLA, and PLATZ-A1. (*A*) Physical interaction between GRF4 and RHT1-GRAS, PLATZ-A1 and empty vector LAW11 (autoactivation test). (*B*) Quantitative alpha-gal Y3H assay between PLATZ-A1 (BD) and RHT1 (AD) with GRF4 expressed or not expressed as the third protein. (*C*) Quantitative alpha-gal Y3H assay between GRF4 (BD) and RHT1 (AD) with PLATZ-A1 expressed or not expressed as the third protein. *** = *P* < 0.001 and ns = not significant. Error bars are SEM based on n = 12. Data in Dataset S10.

DELLA has been reported to physically interact with the GROWTH-REGULATING FACTOR 4 (GRF4) (6), so we evaluated the three-way physical interactions between DELLA, PLATZ1, and GRF4 using yeast-three-hybrid (Y3H) assays. We first determined that GRF4 does not exhibit autoactivation, and that it can interact with DELLA but not with PLATZ-A1 in Y2H assays (Fig. 5*A*). We found that expression of GRF4 as the third protein in quantitative alpha-gal Y3H assays, reduced the interaction between PLATZ1 and DELLA by 74% (Fig. 5*B*, *P* < 0.001). In contrast, when PLATZ-A1 was expressed as the third protein in the Y3H assay, it did not significantly alter the intensity of the interaction between GRF4 and DELLA (*P* = 0.58, Fig. 5*C* and Dataset S10). This result is consistent with the >20-fold stronger interaction between GRF4 and DELLA than between PLATZ and DELLA (Fig. 5 *B* and *C*). Taken together, these results suggest that GRF4 can be a competitor for the DELLA – PLATZ-A1 interaction.

### Natural Variation in *PLATZ-A1*.

To explore if *PLATZ-A1* dwarfing alleles were selected during wheat domestication and breeding, we determined the frequency of *PLATZ-A1* natural variants using available exon capture data from 45 common wheat lines deposited in the Wheat T3 database and genomic sequences available through the PanGenome Project (32). In addition to the 13-bp deletion found in UC1110 and the splice site mutations detected in P515HP, we found two additional deletions in the third exon of *PLATZ-A1.* The first one is a 4-bp deletion in the reference genome of CS RefSeq v1.1 (CAGG deletion between 6A:145,651,864 and 145,651,865, in the - strand) and the second one is a 19-bp deletion (CS RefSeq v1.1, 6A:145,651,892 - 145,651,910). Finally, we detected a 384-bp insertion in the promoter region 130 bp upstream of the start codon in CDC Landmark (CS RefSeq v1.1, 6A:145,654,767 - 145,654,768). The annotated sequence in CDC Landmark indicates a 232-bp insertion relative to CS, but Sanger sequencing and PCR product sizes from CDC Landmark (*SI Appendix*, Fig. S7) confirmed the presence of a 384-bp insertion. The effect of the different mutations on the encoded PLATZ1 protein, together with a haplotype analysis including the *RHT25* natural alleles, is presented in Dataset S11.

We designed a single pair of primers (PlzAF2/PlzAR1, Dataset S2) that can detect all four truncation mutations, as well as a KASP marker that detects the promoter insertion (*SI Appendix*, Fig. S7). We used these primers to screen 46 *T. turgidum* ssp. *dicoccoides*, 78 *T. turgidum* ssp. *dicoccon*, 508 *T. turgidum* ssp. *durum* (Dataset S12), and 1,120 common wheats (Dataset S13). The common wheats included 45 modern spring and winter varieties from the Wheat Coordinated Agricultural Project (WheatCAP), 236 accessions from the North America photoperiod insensitive spring wheat association mapping panel (hereafter referred to as the spring panel), and 839 from the USDA National Small Grains Collections (NSGC, Dataset S13).

The combined *RHT25* mutations represented 30% of the hexaploid accessions, but were completely absent from the ancestral or modern tetraploid wheat accessions investigated in this study (Datasets S12 and S13). The frequency of the combined mutant alleles in hexaploid wheat was significantly higher (χ^2^ tests *P* < 0.001) among the advanced materials (29.7%) than among landraces (5.0%). Within the advanced materials, the mutant alleles were more frequent among those released after 1960 (28.1%) than among those released before 1960 (14.9%, Dataset S13). These results suggest that the *PLATZ-A1* mutant alleles were subject to positive selection during wheat improvement.

The most frequent mutation in the hexaploid wheat collection was the 13-bp deletion (*Rht25b*, 15.8%), followed by the promoter insertion (*Rht25f*, 11.0%), whereas the other three truncation mutations (*Rht25c-e*) were detected in <2% of the accessions (Dataset S13). Both the *Rht25b* and *Rht25f* alleles showed higher frequencies in advanced materials than among landraces (*P* < 0.001), and in materials released after 1960 than before, but the latter was significant only for *Rht25b*. The other truncation alleles showed no significant differences, but the frequencies were too small to compare (Dataset S13).

### Evaluation of the Effect of Natural *RHT25* Alleles on Plant Height.

We used biparental populations segregating for *Rht25c*, *Rht25d*, *Rht25e*, and *Rht25f* to study the effects of these alleles on plant height (Dataset S14). To characterize the effect of *Rht25c* (19-bp deletion) on plant height, we used the previously published population McNeal (*Rht25c*) x Thatcher (*Rht25f*) (33). This population showed a QTL for plant height in the *RHT25* region on 6AS and is fixed for the *Rht24a* allele in 6AL. We genotyped 153 RILs from this population with the *PLATZ-A1* marker and confirmed that *Rht25c* was associated with a highly significant decrease (*P* < 0.001) in plant height relative to *Rht25f* in a factorial ANOVA including the *RHT-D1* gene, which was also segregating in this population (Dataset S14).

To characterize the effect of *Rht25d* (splice mutation), we used the Berkut (*Rht25a*) × P515HP (*Rht25d*) NAM population. We identified an F_5_ line heterozygous for the *PLATZ-A1* region and homozygous for *Rht24b* and derived a heterogeneous inbred family (HIF). We genotyped 62 F_5:2_ HIF lines with the *PLATZ-A1* marker and found a highly-significant effect on plant height associated with the *Rht25d* allele (8.6 cm, *P* < 0.001, Dataset S14).

To study the effect of *Rht25e* (4-bp deletion) on plant height, we evaluated a population from the cross between RSI5_ (_*_Yr5+Yr15+Glu-A1a+GPC-B1_*_)_ (hereafter RSI5, *Rht25f* ) and PI 520033 (*Rht25e*). We identified a single F_4_ plant heterozygous for *RHT25* and fixed for *Rht24a*, *Rht-B1a*, and *Rht-D1a;* and tested its F_5_ progeny for plant height in the greenhouse. The plants homozygous for the *Rht25e* allele were 5.5 cm shorter than those homozygous for the *Rht25f* allele (*P* = 0.009). We also compared BC_4_F_2_ homozygous sister lines from the cross between CS (*Rht25e*, used as recurrent parent) and Spring Hobbit (*Rht25a*). Plants homozygous for the Spring Hobbit allele (*Rht25a*) were 9.6 cm taller than those carrying the *Rht25e* allele (*P* < 0.05).

For *Rht25f* (promoter insertion), we developed one F_3:2_ and two F_4:2_ populations from the cross between CDC Landmark (*Rht25f* ) and Berkut (*Rht25a*). An ANOVA combining the three families and using family as block showed a 2.1-cm reduction in plant height in the sister lines carrying the *Rht25f* relative to the wild type but the difference was marginally not significant (*P =* 0.0613). However, two indirect sources of evidence suggest that this small difference is likely real. First, the differences in plant height between *Rht25f* and truncation mutants *Rht25c* (2.8 cm) and *Rht25e* (5.5 cm) were smaller than between the wild-type allele *(Rht25a*) and the truncation mutations *Rht25b* (17.7 cm), *Rht25d* (8.6 cm), and *Rht25e* (9.6 cm, Dataset S14). In addition, the promoter insertion in *Rht25f* was associated with a 38% decrease in *PLATZ-A1* transcript levels relative to *Rht25a* in the elongating peduncles (*P* < 0.05) in qRT-PCR experiments (Dataset S15).

## Discussion

### *PLATZ-A1* Is the Causative Gene for the *RHT25* Locus.

In this study, we show that among the genes linked to *RHT25* (21), *TraesCS6A02G156600* is the only one with polymorphisms that differentiate tall and semidwarf *RHT25* alleles in two different segregating populations. We also show that induced mutations in *TraesCS6A02G156600* result in reduced height, and that transgenic lines overexpressing this gene partially complement the mutant phenotype. Taken together, these results demonstrate that *TraesCS6A02G156600* is the causative gene underlying the wheat plant height locus *RHT25*.

*TraesCS6A02G156600* encodes a plant-specific PLATZ zinc-dependent DNA-binding protein with two conserved noncanonical zinc finger domains (*SI Appendix*, Fig. S2). This protein was shown to belong to PLATZ group III in a phylogenetic analysis of the wheat PLATZ proteins (26). Our phylogenetic analysis of group III proteins revealed four clusters (*PLATZ1* to *PLATZ4*), each including homologous proteins from wheat, *Brachypodium*, rice, and maize (*SI Appendix*, Fig. S1). This result suggests that the differentiation of these four clusters occurred before the radiation of the major grass subfamilies.

### *PLATZ* Genes from Group III Have Multiple Pleiotropic Effects.

Among the four clusters in PLATZ group III, the related *PLATZ1* and *PLATZ2* clusters are the closest to the well-characterized *PsPLATZ1* from pea (*Pisum sativum*) (25), its related Arabidopsis homolog *AtORESARA15* (29), and a rice gene known as either *GL6* or *SG6* (34, 35) (*SI Appendix*, Fig. S1). Therefore, we compare below the functions of these genes with those observed in this study for wheat *PLATZ1.*

*PsPLATZ1* was the first *PLATZ* gene identified in plants, and its two zinc-finger domains were found to be required for both Zn-binding activity and for binding to A/T-rich DNA sequences (25). Its closest Arabidopsis gene, *AtORESARA15*, was shown to be involved in the regulation of both leaf growth and senescence (29). Loss-of-function mutations in this gene resulted in smaller leaves and accelerated senescence under salt stress, whereas a dominant mutant showed multiple pleiotropic effects including enlarged leaf size, extended leaf longevity, increased plant height and root length, and increased seed volume and weight (29).

Similar effects on seed volume and weight were observed in the rice *PLATZ* gene *Os06g0666100* (also known as *GL6* or *SG6*) (34, 35), which is related to the wheat *PLATZ1* gene (*SI Appendix*, Fig. S1). Rice loss-of-function mutants for this gene have reduced grain length (−10 to −13%) and weight (−14 to −21%) but no significant changes in grain width, whereas transgenic plants with higher *Os06g0666100* expression levels showed significantly longer and heavier grains (34, 35). The wheat grains in the combined *platz1* mutant showed a similar trend, but the effects on grain length (−1.4%) and grain weight (−4.1%, Dataset S4B) were smaller than those observed in rice. The transgenic wheat line overexpressing *UBI::PLATZ1* was male sterile, so we were not able to test the effect of the transgene on grain size; yet we observed a significant increase in glume size (Dataset S7C), similar to what was observed in rice transgenic plants.

The rice *gl6* mutant showed a significant increase in the number of spikelets and grains per panicle relative to the wild type (34). The *Rht25b* dwarfing allele was previously associated with a small increase in spikelet number per spike, but this increase was not translated into a significant increase in grain number per spike (21). This result is consistent with the absence of significant differences in number of grains per spike and grains per m^2^ observed in this study in greenhouse and field experiments, respectively (Dataset S4). The wheat *UBI::PLATZ1* transgenic plants were male-sterile, a phenotype that may have also contributed to the dramatic reduction in grain number observed in the transgenic rice plants overexpressing *Os06g0666100* (34, 35). Similar to the rice *gl6* mutant, the wheat *platz1* mutant showed a reduction in plant height and grain yield. Taken together, these results suggest conserved functions of the *PLATZ* genes from group III in the wheat and rice lineages.

The multiple pleiotropic effects of these *PLATZ* genes parallel their ubiquitous expression profiles in both wheat (*SI Appendix*, Fig. S6) and rice (34, 35). Although transcripts were detected in all tested tissues, expression was the highest in the early stages of inflorescence development in both species. In wheat, *PLATZ1* was also expressed at high levels in the early stages of stem elongation (*SI Appendix*, Fig. S6) and in the elongating peduncle in the nodes and its adjacent region. This result is consistent with the stronger expression of *Sg6* in the nodes of the elongating stems in rice, where cell division contributes new cells to stem elongation (35).

Wheat and rice also showed expression of these related *PLATZ* genes in the grains. In wheat grains, *PLATZ-A1* is the highest in later stages of development (*SI Appendix*, Fig. S6), whereas in rice *Sg6* is highly expressed in the embryos but not in the endosperm. Finally, in situ hybridization of *Gl6* in rice showed expression in stamen primordia and mature anthers (34), which may be related to the male sterility observed in the *UBI::PLATZ1* wheat lines in this study.

### Proposed Action Mechanisms of PLATZ Proteins from Group III.

The shorter spikelet hull and grains in the rice *gl6/sg6* mutants were determined by reduced cell numbers and were associated with the downregulation of multiple cell-cycle-related genes that promote cell proliferation (34, 35). In Arabidopsis, *AtORESARA15* was also found to promote early leaf growth by enhancing the rate and duration of cell proliferation activity. A dominant mutant of this gene (*ore15-1D*) showed an upregulation of genes with positive effects on cell proliferation, such as *Cyclin D3;1*, *GROWTH-REGULATING FACTOR 5* (*GRF5*) and its cofactor *GIF1*; and a downregulation of negative regulators such as miR396, which targets *GRF* transcription factors for degradation. The AtORESARA15 protein was found to directly promote the expression of *GRF1* and *GRF4* (29).

In wheat, we found a similar set of cell cycle-related genes that were significantly down-regulated in the elongating peduncle of the CRISPR *platz1* combined mutant relative to the wild type. These genes include *GRF1*, *GRF5*, *GRF9*, *GRF10*, *GRF11*, and CYCD4;1 (−17 to −55% reduction, *P* < 0.05, Dataset S16). We also observed reduced levels of *GRF3* (−43%, *P =* 0.06), *GRF4* (−30%), *GIF1* (−30%) and cyclins CYCD1;1 (−11%) and CYCD3;1 (−16%, *P* = 0.06) in *platz1*, but those reductions were not significantly different form the wild type (Dataset S16). Taken together, these results suggest that reduced cell proliferation likely contributed to the reduced wheat plant height of the *platz1* mutant, whereas increased cell proliferation contributed to the longer stems, leaves and glumes of the transgenic *UBI::PLATZ1* plants. These results also suggest a conserved role of the *PLATZ* genes from group III in the regulation of cell proliferation.

The physical interaction between wheat PLATZ-A1 and DELLA provides a potential additional mechanism by which PLATZ proteins can affect plant height. The biological relevance of this physical interaction is supported by the genetic interaction between *RHT25* and *RHT1* on plant height. This interaction was previously observed in hexaploid wheat using the natural *Rht25b* allele (21), and confirmed in in this study in tetraploid wheat using the EMS-induced *platz-A1* mutant. These results indicate that *PLATZ1* and *DELLA* operate in a connected pathway regulating plant height. We currently do not know the mechanism by which the PLATZ1-DELLA protein interaction regulates plant height, but three general hypothetical mechanisms are outlined in *SI Appendix*, Fig. S8.

DELLA proteins have a unique N-terminal GA perception region for binding the GA receptor GID1 and a conserved C-terminal GRAS domain required for its repression activity through interactions with multiple regulatory proteins. Mutations in the wheat DELLA N-terminal region in the *Rht-B1b* alleles result in GA-insensitivity and the accumulation of DELLA, which has negative effects on nitrogen-use efficiency and growth (6, 36). Studies in rice have shown that the negative effects associated with the accumulation of DELLA can be counterbalanced by the opposing activity of the *GRF4* transcription factor, which physically interacts with DELLA (6). We observed in wheat a similar interaction between DELLA and GRF4 proteins (Fig. 5*A*), and show that this interaction is able to interfere with the interactions between PLATZ1 and DELLA proteins in Y3H assays (Fig. 5*B*). These results suggest that PLATZ proteins from group III may be involved in the significant role played by the GRF–DELLA interaction on the regulation of plant growth (6).

### *RHT25* Natural Variation and Its Potential for Wheat Improvement.

The natural variants identified in *PLATZ-A1* likely originated in hexaploid wheat, because we were not able to detect them in the wild or cultivated tetraploid wheat species. The *Rht25b*, *Rht25c,* and *Rht25f* alleles were found in hexaploid wheat landraces from Armenia, Iran, Greece, Georgia, and Turkey, indicating a relatively old origin in a region close to the geographical origin of hexaploid wheat. By contrast, *Rht25d* was found only in more modern germplasm, which suggests a more recent origin. For the *Rht25e* allele (present in Chinese Spring), its origin is difficult to infer because it was detected only in a limited number of varieties from diverse locations (Dataset S13).

The significant increases in the proportion of *PLATZ-A1* mutant alleles in advanced materials relative to landraces, and within the advanced materials the increases in post-1960 relative to pre-1960 releases, suggest a positive selection pressure for the mutant alleles in modern wheat-breeding programs. The increased frequency of *platz-A1* mutant alleles in modern materials may have also contributed to the higher frequency of *platz-A1* loss-of-function alleles among semidwarf varieties (42.6%) than among tall varieties (23.1%, Dataset S13). We also observed a significantly higher proportion of *platz-A1* mutations among spring varieties developed under spring planting (71.8%) than among those developed under fall planting (41.7%, Dataset S13). Taken together these results suggest that the natural *platz-A1* dwarfing alleles have an overall positive effect in hexaploid wheat performance, which likely outweighs the negative effect on grain size detected in the induced *platz1* mutants in tetraploid wheat. However, complex interactions between “source” and “sink” traits, and among different “sink” components affect wheat grain yield in a dynamic way across developmental stages (37), and will need to be evaluated to determine the value of deploying the *RHT25* dwarfing alleles in different genetic backgrounds and environments.

One of the motivations for identifying the *RHT25* causative gene was the current interest in wheat-breeding programs to replace the *RHT1* GA-insensitive alleles by GA-sensitive ones to improve seed planting depth, nitrogen use efficiency and biomass production (6, 8, 36). An advantage of the *platz-A1* mutants is that they are already present in adapted germplasm from multiple origins, which can accelerate their deployment in breeding programs. Depending on the optimal wheat plant height for a specific environment, the *platz-A1* mutants can be combined with other GA-sensitive dwarfing genes (e.g., *Rht8*, *Rht18*, etc.) to generate GA-sensitive semidwarf varieties without the *RHT1* dwarfing genes.

In summary, this study reports the identification of *PLATZ-A1* as the causative gene of the GA-sensitive *RHT25* plant height locus and its interactions with DELLA. It also describes natural *platz1* mutant alleles and provides diagnostic molecular markers to study their distribution and accelerate their deployment in wheat-breeding programs. The variable effect of these mutants on plant height can be used to fine-tune wheat plant height in both semidwarf and tall wheat varieties. In combination with other GA-sensitive dwarfing alleles, the *platz1* mutants can be also used to develop GA-sensitive semidwarf varieties and explore their potential to increase grain yield beyond the GA-insensitive semidwarf varieties of the Green Revolution.

## Materials and Methods

The mapping populations used in this study to characterize the different *RHT5* alleles are described in *SI Appendix*, *Method S1*. The methods for the phylogenetic analysis of PLATZ group III proteins are described in *SI Appendix*, *Method S2*, whereas the EMS-induced, CRISPR-induced, and natural *PLATZ1* mutants are described in *SI Appendix*, *Methods S3–S5*, respectively. Seeds of the Kronos *platz-A1 platz-B1* combined mutations backcrossed four times to Kronos (BC_4_F_3_) were deposited under the identification number PI 702421 into GRIN-Global (https://npgsweb.ars-grin.gov/gringlobal/search).

The methods used for plant transformation and subcellular localization of PLATZ-A1 are described in *SI Appendix*, *Method S6*, whereas those used for yeast-two- and yeast-three-hybrid methods are in *SI Appendix*, *Method S7*. Protein Coimmunoprecipitation assays and western blotting methods are in *SI Appendix*, *Method S8*, and RNA extraction and real-time qRT-PCR methods are in *SI Appendix*, *Method S9*. *SI Appendix*, *Method S10* describes the field experiment used to test the effect of *RHT25* on grain yield.

All genome coordinates and identification numbers for the annotated genes are from CS RefSeq v1.1 (38) unless indicated otherwise. Exome sequencing data were extracted from the T3/Wheat database project 2017_Wheat-CAP_UCD (https://wheat.triticeaetoolbox.org/search/genotyping_data_projects).

## Supplementary Material

Appendix 01 (PDF)

Dataset S01 (XLSX)

Dataset S02 (XLSX)

Dataset S03 (XLSX)

Dataset S04 (XLSX)

Dataset S05 (XLSX)

Dataset S06 (XLSX)

Dataset S07 (XLSX)

Dataset S08 (XLSX)

Dataset S09 (XLSX)

Dataset S10 (XLSX)

Dataset S11 (XLSX)

Dataset S12 (XLSX)

Dataset S13 (XLSX)

Dataset S14 (XLSX)

Dataset S15 (XLSX)

Dataset S16 (XLSX)

## Data Availability

Seeds of the *platz1* mutant have been deposited in GRIN-global (PI 702421) (39). All other data used in this study are included in the article and/or *SI Appendix*.
